# UCP2 and pancreatic cancer: conscious uncoupling for therapeutic effect

**DOI:** 10.1007/s10555-023-10157-4

**Published:** 2024-01-09

**Authors:** Emily G. Caggiano, Cullen M. Taniguchi

**Affiliations:** 1 Department of Radiation Oncology, The University of Texas MD Anderson Cancer Center, Houston, TX USA; 2 The University of Texas MD Anderson Cancer Center UTHealth Houston Graduate School of Biomedical Sciences, The University of Texas MD Anderson Cancer Center, Houston, TX USA; 3 Department of Experimental Radiation Oncology, The University of Texas MD Anderson Cancer Center, Houston, TX USA

**Keywords:** Pancreatic cancer, Metabolism, Mitochondria, Uncoupling, Therapeutics

## Abstract

Pancreatic cancer has an exaggerated dependence on mitochondrial metabolism, but methods to specifically target the mitochondria without off target effects in normal tissues that rely on these organelles is a significant challenge. The mitochondrial uncoupling protein 2 (UCP2) has potential as a cancer-specific drug target, and thus, we will review the known biology of UCP2 and discuss its potential role in the pathobiology and future therapy of pancreatic cancer.

## Pancreatic cancer: an aggressive cancer dependent on mitochondrial metabolism

Pancreatic ductal adenocarcinoma (PDAC) is a lethal cancer with a 5-year survival rate of less than 11% [1]. The disease is potentially curable with surgery, but 80–85% of cases are already locally advanced or distantly metastatic at the time of diagnosis, making most cases ineligible for operation. This is exacerbated by the lack of identified biomarkers and the fact that PDAC has such a low incidence in the population that general screening of the population is not justified [2]. Overall, this results in late diagnoses in the majority of PDAC patients. In addition, the main driver and initiator of PDAC, *KRAS* (specifically the G12D mutation)*,* present in over 90% of PDAC cases, is currently undruggable [3], resulting in a dearth of targeted therapies and reliance on powerful nonspecific chemotherapies that are highly toxic to patients. While exciting steps have been made with the FDA approval of the G12C inhibitor sotorasib [4–6], it is not a common mutation in PDAC, present in only 1% of cases [3]. Finally, PDAC exhibits resistance to a large array of treatments. Gold standard agents such as gemcitabine, nab-paclitaxel, and FOLFIRINOX are the most effective compounds but are limited by toxicity and moderate survival benefits [7, 8]. The combination of late diagnosis, lack of targeted therapies, and drug resistance makes PDAC one of the deadliest cancers and the focus of many research groups.

Like many cancers, PDAC also has rewired metabolism [9, 10]. This includes a shift towards aerobic glycolysis, increased lactate production, induction of non-oxidative pentose phosphate pathway, increased fatty acid synthesis, reliance on glucose to grow, and dependency on glutamine and collagen to replenish tricarboxylic acid (TCA) cycle intermediates [9, 11, 12]. While PDAC utilizes aerobic glycolysis, several groups have demonstrated that pancreatic cancer has sufficient metabolic plasticity to also use mitochondrial oxidative phosphorylation (OXPHOS) to generate ATP [13, 14], driving metastasis and treatment resistance [11, 13, 15–17]. While targeting mitochondrial respiration has had some preclinical promise [13, 18], the nonspecific poisoning of mitochondria has failed to demonstrate clinical success due to its suboptimal therapeutic ratio from its high toxicity in normal cells that rely on these same pathways. For instance, the targeting of complex I with the small molecule IACS-010759 showed efficacy against brain cancer and acute myeloid leukemia (AML) in preclinical models by inhibiting proliferation and inducing apoptosis, but the ensuing phase I clinical trial, using relapsed AML and a variety of solid tumors, including 3 PDAC patients, was halted due to modest efficacy and induced resistance with the inability to escalate dose or intensify scheduling due to neurotoxicity [19]. This was also shown with the complex I inhibitor metformin, which failed to improve the survival of PDAC patients when combined with chemotherapy in clinical trials [20].

Despite the difficulty in selectively targeting mitochondrial function, these organelles play a vital role in metabolic reprograming in PDAC via OXPHOS functionality. In particular, the SLC25 family of transmembrane anion carriers mediates glutamine and aspartate homeostasis, which are critical components of the metabolic rewiring that occurs in PDAC. Glutamine enters the mitochondria and is metabolized to glutamate, which is then metabolized to α-ketoglutarate. Thus, glutamine is vital to replenishing TCA cycle intermediates. Further, glutamine is metabolized to aspartate, which is vital for protein synthesis, nucleotide synthesis, fatty acid synthesis, and reactive oxygen species (ROS) elimination [16], all important pathways in PDAC. However, these two integral components of PDAC tumorigenesis and progression cannot traverse the mitochondria alone and thus need carriers to transport them across the inner mitochondrial membrane. UCP2’s proposed role as a metabolite transfer protein may be indispensable to PDAC growth, in addition to its other functions, as is covered below.

As a result, many of the SLC25 family members are dysregulated in PDAC, UCP2 among them [21]. Moreover, UCP2 levels are increased in leukemia and cancers of the ovary, bladder, esophagus, testes, kidneys, lungs, colon/rectum, and prostate [22, 23]. This overexpression has been correlated to chemotherapeutic agent resistance in leukemia as well, with drug-resistant cell lines significantly overexpressing UCP2 compared to sensitive cell lines [24]. In addition, UCP2 overexpression has been shown to reduce ROS levels and apoptosis upon chemotherapeutic exposure, thus promoting drug resistance in colon cancer [25].

## The SLC25/mitochondrial uncoupling protein family

To better understand how UCP2 is involved in the pathobiology of pancreatic cancer, it would be relevant to broadly review the biology of the family of proteins. UCP2 is a member of the SLC25 mitochondrial anion transporter family [26]. These proteins, also known collectively as the mitochondrial carrier family, make up the largest group of solute carriers. Located mainly in the mitochondria with varying distribution of tissue expression between the family members, they have an overarching function to link the metabolic functions of the cytosol and the mitochondria by providing the transport of a wide variety of solutes across membranes. The main features connecting all members of the SLC25 family and making them a unique and distinguishable group are a tripartite structure, a threefold repeat signature motif, and 6 transmembrane alpha helices [26].

The UCP subfamily consists of 6 members (UCP1-6), all residing as integral membrane proteins in the inner mitochondrial membrane [27, 28]. All family members uncouple the ETC proton gradient from ATP generation by allowing protons to “leak” or flow back down their gradient into the inner membrane space. Each protein has different tissue expressions and homology to each other. Generally, UCP1 is exclusively expressed in brown adipose tissue (BAT), UCP3 is mostly contained to human skeletal muscle and rodent skeletal muscle and BAT, UCP4/5 is limited to neuronal expression, and UCP6 is only expressed in kidney tissue. In contrast, UCP2 is expressed ubiquitously. In addition, there is large homology between the proteins and between mouse and human homologs.

One of the most abundant and the classic member of the UCP2 subfamily is Uncoupling Protein 1 (UCP1), making up to 15% of mitochondrial protein mass [29]. UCP1 was identified in 1978 [30] and first cloned in 1986 [29]. It is expressed exclusively in BAT and is classically involved in mediating the non-shivering thermogenesis of this tissue; thus, its uncoupling generates heat, protecting against cold and controlling energy expenditure. Mice lacking Ucp1 are sensitive to cold and are unable to maintain body temperature in cold temperatures [31].

UCP2 was first discovered and cloned in 1997 [32, 33]. Due to its homology to UCP1 (about 59%), as well as its chromosomal location near regions linked to obesity and hyperinsulinemia and its ability to uncouple, UCP2 was first thought to also be involved in thermogenesis [32]. However, mice lacking Ucp2 were still able to maintain body temperatures in cold temperature, as well as body fat levels [34]; thus, UCP2 was unlikely to be involved in thermogenesis like UCP1. UCP2 is located on chromosome 7 of the mouse and chromosome 11 of humans, spanning 8.4 kb over eight exons and 7 introns. Upon transcription, it contains two untranslated exons followed by six coding exons [35]. In mice, Ucp2 is expressed in most tissues, and prominently in the lung, kidney, pancreas, adipose tissue, muscle, heart, brain, and spleen [28, 32, 33, 36, 37]. Human and murine UCP2 display 95% homology, whereas UCP1 and UCP3 are only 58% and 71% homologous to UCP2, respectively.

The SLC25 family members all contain a tripartite structure, three tandemly repeated homologous domains composed of about 100 amino acids, and six transmembrane helices (numbered H1 to H6), connected with two loops on the cytosolic side and three on the matrix side. Both the N and C termini are orientated toward the intermembrane space. These helices form a barrel that forms a depression accessible to the outside [38]. The prolines of the odd numbered helices form a sharp kink, in known conserved regions of SLC25 mitochondrial transporter family members [26]. Charged residues form a salt bridge network that connects the transmembrane helices, closing the matrix side of the channel [39]. In addition, the SLC25 family has a single binding site that is only exposed to one side of the matrix at a time, depending on its conformation in the membrane. It is in the c-state when the substrate enters from the cytosolic side and the m-state when it enters from the matrix side, with a transition state in between. In the transition state, the substrate is deep in the center of the carrier, with both sides mostly closed off to either side of the membrane [26]. The structure of UCP2 was recently determined utilizing nuclear magnetic resonance [38]. The structure resembles that of the ADP/ATP carrier adenine nucleotide translocase (ANT), with conserved proline kinks and helices, but the third tandem repeat is significantly different than the ANT when bound to their respective inhibitor [38]. This difference results in a matrix side that is significantly more open in UCP2, resulting in a less-obstructed channel compared to ANT.

## Dual roles for UCP2: an uncoupler and a transporter

### Mechanisms of uncoupling

**Fig. 1:**
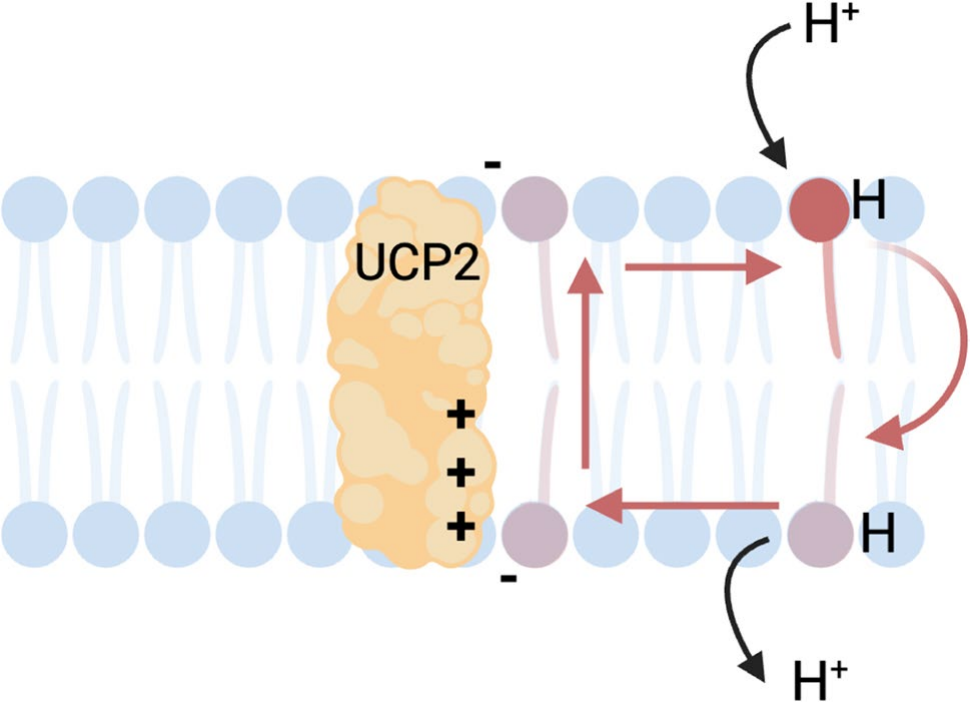
The fatty acid protonophore flip-flop mechanism of Ucp2. Fatty acids bind a proton and flip to the other side of the membrane. After the proton has disengaged from the fatty acid, the anionic fatty cannot flop back across the membrane. The negatively charged fatty acid is electrostatically attracted to positively charged amino acid residues in Ucp2, which then assists in moving the fatty acid back to the original side of the membrane so that it can bind another proton and repeat this process

As its name implies, UCP2 was first identified as an uncoupling protein, allowing protons to flow back down the gradient generated during the ETC, without generating ATP, thus uncoupling the proton gradient from the ATP generation. There were two debated mechanisms of how UCP2 went about its uncoupling capabilities: the fatty acid (FA) cycling mechanism and the FA shuttling mechanism [39]. The FA cycling mechanism was proposed by Fedorenko et al. and maintains that UCP2 is a carrier of FA, whereby FAs bind a proton, travel through UCP2 in the protonated form, release the proton on the other side of the membrane, and travel back through UCP2 in the anionic form [40]. At first, studies demonstrating that histidine residues vital to proton transport in UCP1, H145, and H147, were not present in UCP2, generating support for the FA shuttling mechanism [41]. However, other studies showed that UCP2 has ample basic residues that can take the place of these histidines [42], and multiple studies disproved the FA cycling mechanism (reviewed in [39]). It is now agreed that UCP2 acts in a FA cycling capacity, otherwise known as the protonophoretic model, and involves a flip-flop mechanism [43–45]. In this model, protons bind to FA head groups on the outer leaflet of the intermembrane side of the mitochondrial membrane. The FA then flips across the membrane and releases the proton into the matrix. However, the FA becomes anionic in the process and is thus unable to flop back to the other side of the membrane unaided. The negatively charged FA now becomes electrostatically attracted to the positively charged amino acid residues Arg60 and Lys271 present in UCP2. UCP2 can then flip the FA back, where it continues binding protons (Fig. 1) [46]. In this mechanism polyunsaturated FAs are the most potent UCP2 activators because their double bonds increase the disorder and fluidity of the membrane, thus the FAs are able to move and reorient faster than that of the more rigid membrane seen with saturated FAs [43, 47]. This uncoupling mechanism has been supported by multiple studies showing that overexpression of UCP2 in yeast [32] or proteoliposomes containing UCP2 [42, 48] induced proton flux and that thymocytes from mice lacking Ucp2 had reduced proton leak compared to wild-type mice [49].

Despite the evidence that UCP2 is an uncoupling protein, multiple groups have also determined that UCP2 does not have uncoupling abilities. Embryonic fibroblasts lacking Ucp2 reduced fatty acid oxidation, leading to an increase in glucose metabolism through glycolysis, likely to maintain levels of acetyl-CoA. In addition, they exhibited an increase in proliferation and a reduction of NADPH, all without a change in ROS levels or evidence for a role of uncoupling [50]. Ucp2 loss *in vivo* also had no impact on the inner mitochondrial membrane’s permeability to protons, nor was there an increase in the ATP/ADP ratio as one would expect with the loss of an uncoupling protein in the spleen and lung mitochondria. A potential issue with this finding is that the experiments were conducted using isolated mitochondria, thus additional factors needed to induce uncoupling may have been lost [51]. In addition, Bertholet et al. did not find evidence to support UCP2 to be the FA-dependent mediator of proton leak in the mitochondria, instead identifying the ATP/ADP carrier as the likely candidate [52]. Finally, Robson et al. generated a β-cell-specific knockout mouse but did not find any impact on rates of uncoupling respiration and ATP content [53]. Although these results do not support UCP2 as an uncoupling protein, they may give evidence to the fact that UCP2 likely has functions other than an uncoupling protein, such as a metabolite transfer protein, potentially depending on the tissue and environment. Other reasons for lack of uncoupling ability may stem from its low endogenous levels in the cell, or that activation is required.

### Reactive oxygen species regulation

UCP2’s relationship with controlling ROS levels has been well established. UCP2 was first linked to ROS in the same year it was discovered, when GDP inhibition of the protein resulted in an increase in hydrogen peroxide, similar to that of UCP1. It was concluded that UCP2 modulates ROS levels via membrane potential alterations, as these effects were not seen in UCP2 null hepatocyte cells [54]. When UCP2 was lost in pancreatic β-cells, superoxide levels were significantly increased, contributing to the β-cell dysfunction in obesity and diabetes [55], and overexpression of UCP2 in pancreatic β-cells protected against ROS-induced toxicity [56] suggesting UCP2 is involved in antioxidant defense in these cells. Loss of UCP2 via antisense oligonucleotides in endothelial cells also resulted in increased levels of intracellular ROS and oxidative stress [57]. Ucp2 loss in mice resulted in increased ROS species [34], as well as oxidative stress, with upregulation of multiple antioxidant genes and impaired glucose-stimulated insulin secretion [58]. Mice without Ucp2 had elevated staining of nitrotyrosine in islets, an *in vivo* marker for peroxynirite, thus suggesting local oxidative stress in these tissues [58]. To avoid limitations of the whole-body knockout model of Ucp2 used previously, β-cell-specific Ucp2 knockout mouse were generated, and intracellular ROS levels were increased, as well as glucose-stimulated insulin secretion, and aberrant glucagon secretion [53]. This role in ROS regulation is vital to cell health, as dysfunction in ROS control can lead to a wide array of pathologies, including β-cell dysfunction and diabetes.

### Metabolite transfer functions

In addition to uncoupling, UCP2 has been implicated in metabolite transfer properties. When reconstituted in lipid vesicles, UCP2 exchanged malate, oxaloacetate and aspartate for phosphate and a proton across the inner mitochondrial membrane [59], reducing the levels of these C4 metabolites in the mitochondrial matrix. This potential function resulted in decreased oxidation of glucose, leading to reduced acetyl-CoA generation and redox pressure, as well as minimizing the ATP to ADP ratio and ROS production [59]. In addition, it enhanced glutaminolysis in the matrix of the mitochondria, resulting in significantly more C4 metabolites exported to the cytosol [59]. In PDAC, glutamate-derived aspartate is crucial for generation of reduced glutathione [12] to control ROS levels. Thus, UCP2’s export function is vital for the maintenance of PDAC’s dependence on glutamine, redox control, and aerobic glycolysis state [59]. Ucp2 knockout in a skin carcinogenesis model supported the function of Ucp2 as a metabolite transfer protein, as the changes in pyruvate, succinate, and malate levels seen in wild-type animals were lost in knockout mice [60]. UCP2 overexpression was shown to shift cancer cells away from glycolysis and back towards the normal pancreatic tissue phenotype of OXPHOS utilization, reducing tumorigenicity [61] without impacting membrane potential. In addition, UCP2^−/−^ macrophages had impaired glutamine metabolism and reduced accumulation of aspartate, but no impact on mitochondrial uncoupling or the functional state of the mitochondria, suggesting that the glutamine metabolism role of UCP2 is independent of its uncoupling ability [62]. Finally, UCP2 was identified as the aspartate exporter out of the mitochondria upon metabolism of glutamine in PDAC cells. UCP2 loss significantly impacted growth of PDAC tumors *in vitro* and *in vivo* but only in cells with the KRAS mutation characteristic of PDAC [16]. UCP2 loss coupled with KRAS mutation to decrease the growth of PDAC cells and cause aberrant redox homeostasis, in addition to reducing glutaminolysis. In KRAS WT cells, only glutaminolysis was impacted, demonstrating the connection between UCP2 and the rewired metabolism in PDAC [16].

### Calcium transporter functions

Recent findings have also identified UCP2 in mitochondrial calcium (Ca^2+^) transport. UCP2 was first linked to Ca^2+^ in 2007 by Trenker et al. Utilizing overexpression, mutagenesis and knock down studies, they determined that UCP2 is required for Ca^2+^ uptake in cells. While overexpression of either protein did not affect release of Ca^2+^ from the endoplasmic reticulum, or basal concentrations in the mitochondria and endoplasmic reticulum, uptake, and sequestration of Ca^2+^ was significantly increased [63] This was not seen with UCP1, suggesting a unique physiological function for UCP2. In addition, when UCP2 was reduced via siRNA, mitochondrial Ca^2+^ uptake was reduced. Finally, they identified a domain homologous in UCP2 that is distinct from UCP1 responsible for Ca^2+^ uptake: the intermembrane loop 2. Upon mutation of this region, UCP2 correctly localized to the mitochondria, but overexpression did not increase sequestration of Ca^2+^ as seen with the wild-type protein [63].

Later, it was determined that the role of UCP2 depends on the source of the Ca^2+^. While Ca^2+^ mobilized specifically from the endoplasmic reticulum was preferentially impacted by UCP2 function, Ca^2+^ entering the cell by crossing the plasma membrane (otherwise known as store-operated calcium entry, SOCE [64]) was UCP2 independent [65]. Knockdown experiments demonstrated that loss of UCP2 only impacted Ca^2+^ release from the endoplasmic reticulum, showing their role in uptake from specific routes of Ca^2+^ sources. However, overexpression of the protein increased Ca^2+^ sequestration from both sources, indicating that mitochondria uptake their Ca^2+^ via distinct pathways that depend on the Ca^2+^ source [65]. It is discussed that there must be UCP2-independent mechanisms of mitochondrial Ca^2+^ uptake, but it was unknown at this time what this pathway may be.

It was determined in 2011 that the main route of Ca^2+^ influx is via the mitochondrial calcium uniporter (MCU) [66, 67], and identification of other proteins involved in Ca^2+^ flux across the inner mitochondrial membrane soon followed, including mitochondrial Ca^2+^ uptake 1 (MICU1) and essential MCU regulator (EMRE) [68]. While MCU, MCUb (a dominant negative subunit), and EMRE form the pore that Ca^2+^ traverses, MICU1 and MICU2 regulate the uptake of Ca^2+^ into the mitochondria and prevent overload [69]. The identification of the units involved in Ca^2+^ allowed for elaboration in the specific role UCP2 plays in Ca^2+^ flux in the mitochondria. Madreiter-Sokolowski et al. determined that protein arginine methyltransferase 1 (PRMT1) regulates MICU1 via methylation at position 455 [70], reducing its Ca^2+^ sensitivity and uptake. However, in the presence of UCP2, which binds specifically to methylated MICU and abolishes its sensitivity to PRMT1, leading to re-establishment of normal Ca^2+^ import function [70]. This was reiterated by Gottschalk et al. utilizing super-resolution microscopy, who also reported that UCP2 anchors MCU to the inner boundary membrane of the mitochondria after ER Ca^2+^ release [71]. In summary, UCP2 plays an important role in ER Ca^2+^ release, sequestration of Ca^2+^ in mitochondria, and regulation of Ca^2+^ flux into cells via MCU.

### Controversies over mechanisms of UCP2 activation

The mechanism of UCP2 activation is largely debated. UCP2 has low endogenous expression in tissue and thus is likely activated to achieve physiologically relevant levels. It was first determined in 1999, 2 years after its initial discovery, that FA is required for proton flux of UCP2, while purine nucleotides inhibit it [42]. In addition, UCP1 utilized CoQ as a required cofactor [72], so it was explored whether UCP2 acts in a similar fashion to the original uncoupling protein. In 2001, Echtay et al. determined that UCP2 has uncoupling activity and that coenzyme Q (CoQ) is required for the transport of protons. In addition, they confirmed that FA is required for UCP2 uncoupling and that nucleotides inhibit this function [48], much like that of UCP1. In 2002, Echtay et al. determined that UCP2 is activated by ROS [73] on the matrix side of the mitochondria [74], then the following year, determined that the lipid peroxidation byproduct 4-hydrpxy-2-noneal (4-HNE) also induces UCP2’s uncoupling abilities [75]. While their confirmation of FA activation was largely accepted and replicated, their other claims have fallen into question.

The requirement of FA, specifically polyunsaturated fatty acids (PUFAs) and the inhibiting actions of purine nucleotides were supported via proteoliposome experiments. FAs such as oleic, myristic, and PUFAs induced the fastest proton flux, followed by PUFAs, but PUFAs had the highest affinity to UCP2 compared to other FAs [76]. In addition, proton flux was inhibited by up to 50% by the presence of ATP, which had the highest affinity for the protein, followed by GTP, GDP, and AMP with the lowest affinity of the purine nucleotides [76]. In addition, planar lipid bilayers have shown that UCP2 has protonophoric function exclusively in the presence of FAs and that polyunsaturation is more effective than saturation of FAs [45]. iPLA2γ, a phospholipase that cleaves the ester bond of membrane phospholipids, resulting in the release of both polyunsaturated and saturated FAs, is activated by hydrogen peroxide in lung and spleen tissues, thus providing a mechanism for UCP2 activation: iPLA2γ increases the levels of free FAs for UCP2 activation, resulting in a reduction in ROS. Thus, iPLA2γ and UCP2 coordinate to reduce oxidative stress in tissues where UCP2 is highly expressed [77]. This mechanism functions the same way in pancreatic β-cells, providing protection from lipotoxicity resulting from free FA-mediated oxidative stress [78]. Furthermore, support for FA binding, especially via the protonophore model, was enhanced when a FA binding site was discerned in UCP2. NMR determined that FA binds to a peripheral site of UCP2 between helices H1 and H6, and partially H2 and H5, in a hydrophobic groove [46]. Through mutagenesis studies, it was determined that residues Arg60 and Lys271 are vital for FA binding to UCP2, providing positive charges for the basic/negatively charged fatty acid head group to interact with on the matrix side of the protein. If both these residues are mutated to neutral amino acids, FA cannot bind UCP2, and proton shuttling is decreased [46].

The inhibitory action of purine nucleotides on UCP2 was inferred to be similar to that of UCP1 and UCP3 because the seven residues involved in inhibition are conserved between the proteins [42]. However, there are large differences in nucleotide sensitivity between the proteins. The mechanism was elucidated following the reveal of UCP2’s structure while it was in complex with GDP. GDP binds deep within the channel of UCP2 [38], in contrast to UCP1, where its inhibitor molecule ATP binds midway in the channel [79]. The binding of GDP induces a conformation change in UCP2, affecting residues Gly281 and Gly19, resulting in the displacement of any bound FAs, preventing further FA binding, and resulting in the loss of UCP2 uncoupling [46]. Residues Arg185, Arg88, and Lys141 within the helices of UCP2 participate in charge-charge binding with GDP to facilitate interaction [38].

While Murphy et al. confirmed that UCP2 was activated by both ROS and 4-HNE, they were in the minority for confirming activation by both species. They proposed that UCP2 is activated indirectly by ROS, specifically superoxide, which is dismutated to hydrogen peroxide by superoxide dismutase and reacts with ferrous iron released via the Fenton reaction to generate the highly reactive hydroxyl radical. This radical then interacts with FA and generates a fatty acyl radical, which initiates lipid peroxidation reactions, leading to the generation of byproducts, including 4-HNE, which activates UCP2 [80]. In contrast, another group found evidence of ROS activating UCP2 and UCP3, but not 4-HNE [81], which was supported by Malingriaux et al. who determined that 4-HNE does not directly activate UCP2, but rather 4-HNE helps increase the proton flux mediated by FA [82]. Endogenously produced superoxide activated UCP2 in pancreatic β-cells, reduced ATP levels and impaired glucose-stimulated insulin secretion [55]. Couplan et al. were unable to replicate the ROS-induced activation of isolated spleen and lung mitochondria in their studies [51], further questioning the validity of the original experiments.

## Regulation of UCP2 expression

### Transcriptional

UCP2 is regulated transcriptionally via transcription-binding sites for specific protein-1 (Sp1), sterol regulatory elements (SRE), thyroid hormone response elements (TRE), and the E-box binding sites in the promoter. It was first established that long-chain FAs can activate transcription of UCP2, but the mechanisms involved was unknown [83]. Both saturated and unsaturated FAs, including palmitic acid, oleic acid, and linoleic acid, were found to directly interact with peroxisome proliferation-activated receptor (PPAR)γ and PPARα [84] and PPARγ agonists were shown to increase UCP2 mRNA levels [85, 86]; thus, these genes were explored for their ability to activate UCP2 via fatty acids. Medvedev et al. determined that the E-box is necessary for promoter activity but PPARγ does not bind this region, thus its stimulation of UCP2 must be indirect via additional transcription factors interacting with the E-box region [87], but the exact factors involved are still unknown. PPARα also stimulates UCP2 expression. In the heart, free fatty acids and the PPARα agonist WY-14643 increased UCP2 expression [88]. In the liver, induction of UCP2 by PPARα was found to lower mitochondrial hydrogen peroxide produced during fatty acid oxidation, thus reducing drug-induced hepatotoxicity [89].

UCP2 expression is regulated by a variety of other factors in addition to PPARs. In endothelial cells, the AMP-activated kinase (AMPK) activator AICAR inhibits palmitate-induced apoptosis by reducing ROS levels, and UCP2 inhibition by GDP reverses this effect [90]. UCP2 overexpression also inhibited apoptosis and ROS generation, suggesting that AMPK inhibits palmitate-induced apoptosis in endothelial cells, and UCP2 may be a mediator [90]. Medvedev et al. determined that in pancreatic β cells SREBP1c, a controller of lipid homeostasis binds to UCP2 via its SRE region and upregulates its expression, potentially via a PPAR-dependent pathway, due to the fact that FAs are natural ligands for these factors [91]. The SRE region is also critical for UCP2’s upregulation by FAs, as deletion of this region negated the response of UCP2 to FA induction [91]. In human pancreatic β cells, the two E-boxes are vital for stimulation of UCP2 promoter activity via the sterol regulatory element-binding protein 1c (SREBP1c), as the SRE region is not conserved in humans [92]. UCP2 is also activated by the cAMP-PKA cascade, resulting in enhanced depolarization of membrane potential after stimulation with cAMP, which was not seen in functional mutant UCP2 neural cells [93]. Treatment with a protein kinase A (PKA) inhibitor also abolished the effects on membrane potential, suggesting that the cAMP-PKA pathway stimulates UCP2 expression in neural cells [93]. PGC-1α, implicated in insulin release by pancreatic β-cells, has been shown to stimulate human UCP2 expression via two thyroid hormone response elements in the promoter [92], and coordinated upregulation of pancreatic Ucp2 and Pgc-1α expression has been shown in animal models of type 2 diabetes [94]. In addition, PGC-1α can indirectly stimulate UCP2 expression via liver X receptor-mediated expression of SREBP1c [92], while PGC-1β can directly activate SREBPs and upregulate UCP2 in pancreatic β cells [95, 96], thus supporting a role for both PGC-1α and β in insulin secretion regulation. The forkhead box protein A1 (FOXA1) transcription factor also interacts with UCP2, identified by a reduction in ATP synthesis in FOXA1^-/-^ pancreatic β cell islets due to an upregulation of UCP2 by Vatamaniuk et al. FOXA1 downregulates UCP2 transcription, potentially through interaction with the UCP2 repressor Sirtuin 1 (Sirt1) [97] as it binds near FOXA1 on the UCP2 promoter [98]. Finally, transforming growth factor beta (TGFβ)-induced SMAD4 was shown to bind to six repressive SMAD binding elements of the UCP2 promoter in breast cancer, repressing UCP2 transcription [99]. SMAD4 is a vital tumor suppressor gene that is inactivated in multiple cancer types, including over 50% of PDAC cases, potentially contributing to the overexpression of UCP2 often seen in these pathologies [100].

### Translational

UCP2 translation is regulated by a myriad of miRNAs, many of which are dysregulated in PDAC [101, 102]. This translational regulation results in protein expression patterns that do not always match mRNA levels. mir-15a, considered a tumor suppressor miRNA that is downregulated in PDAC, negatively regulates UCP2 translation, resulting in increased insulin biosynthesis in pancreatic β cells [103]. Another tumor-suppressive miRNA downregulated in PDAC is mi133a, a muscle-specific miRNA involved in myogenic differentiation [104]. On the other hand, miR-214 is upregulated in PDAC, resulting in increased UCP2 expression. It was first identified by its reduction of oxidative stress associated with diabetic neuropathy via UCP2 overexpression [105]. In addition, miRNAs can indirectly impact PDAC via UCP2, such as with miRNA-2909. miR-2909 suppresses the Kruppel-like factor 4 (KLF4) gene, a tumor suppressor downregulated in PDAC [106]. KLF4 negatively regulates UCP2 expression [107], thus when KLF4 is suppressed via miR-2909, UCP2 expression increases. Other miRNAs targeting UCP2 include miR30e, a recently found inhibitor of PDAC carcinogenesis and progression that acts via restraint of SNAIL-mediated epithelial-mesenchymal transition. miR30e is downregulated in PDAC [108], contributing to the upregulation of UCP2 characteristic in PDAC, as well as that seen in kidney fibrosis [109]. Finally, miR24 and miR-34 also downregulate UCP2 expression [110]. miR-24 is downregulated in PDAC [111] as it inhibits progression via downregulation of Laminin beta subunit 3 (LAMB3), a known oncogene upregulated in PDAC [112]. miR-34 is also downregulated, blocking tumor growth by inhibiting multiple oncogenic signaling pathway genes [113].

In addition to miRNAs, UCP2 translation is altered by glutamine, inducing UCP2 expression at physiological levels [114]. This was supported by the finding that removal of glutamine from the cell media reduced UCP2 protein levels, but not mRNA, supporting a translational impact of glutamine on UCP2 [115]. Hurtaud et al. determined that the upstream open reading frame of the 5′ UTR is required for this action of glutamine [116].

UCP2 translation is also regulated by the RNA-binding protein heterogeneous nuclear ribonucleoprotein-K (hnRNP-K). One study determined that hnRNP-K binds to the UCP2 3′ UTR specifically with insulin treatment of cells, suggesting that hnRNP-K is involved in the insulin-induced translation of UCP2 [117]. Additionally, Angiopoetin-1 stimulates interaction of Src with hnRNP-K leading to the phosphorylation of hnRNP-K and the upregulation of UCP2 translation in endothelial cells, providing an explanation of the quick response of endothelial cells to oxidative stress [118]. In the liver, adiponectin transiently increases ROS levels, initiating the translocation of hnRNP-K, resulting in the upregulation of UCP2 expression and protection against fatty liver diseases and hepatic injury [119].

### Post-translational

UCP2 is negatively regulated post-transcriptionally by reversible glutathionylation [81]. Glutathionylation is stimulated by reduced ROS levels and reversed with increasing ROS levels, creating an on-off switch of UCP2 activity. The protein family GRx is involved in the reaction, and specifically, reactive cysteine residues are the sites of glutathionylation in UCP2. While Cys^259^, located in a mitochondrial matrix-facing loop of the protein, was identified as the main residue involved for UCP3 glutathionylation, specific cysteine residues in UCP2 have not yet been evaluated [81]. This mechanism is also connected to UCP2’s role in negative regulation of glucose-stimulated insulin secretion (GSIS), where an increase in specifically matrix ROS results in deglutathionylation and activation of UCP2, consequently impeding GSIS in pancreatic islet cells [120].

### Protein stability

UCP2 translation is constantly suppressed; thus, the protein has an unusually short half-life. Compared to UCP1 with a half-life of about 30 h, UCP2 is much more unstable, with a half-life as low as 30–60 min in certain tissues [121], including pancreatic β cells [115]. This is likely due to the different physiological functions of the proteins. While UCP1 is involved in the long-lasting thermogenesis process and must be present for long periods of time, UCP2 is involved in ROS levels and changes in nutrient supply, which are tightly regulated and change quickly, thus the proteins involved are synthesized rapidly but are only needed for short periods of time. UCP2 is stable in isolated mitochondria [115]; thus, there must be an extramitochondrial factors involved in UCP2 degradation. It was determined that the degradation of UCP2 is controlled by the cytosolic proteasome [122]; however, the exact mechanism is unknown, as there is no known mitochondrial export machinery in mammalian cells.

## UCP2 as a Therapeutic Target in PDAC

**Fig. 2:**
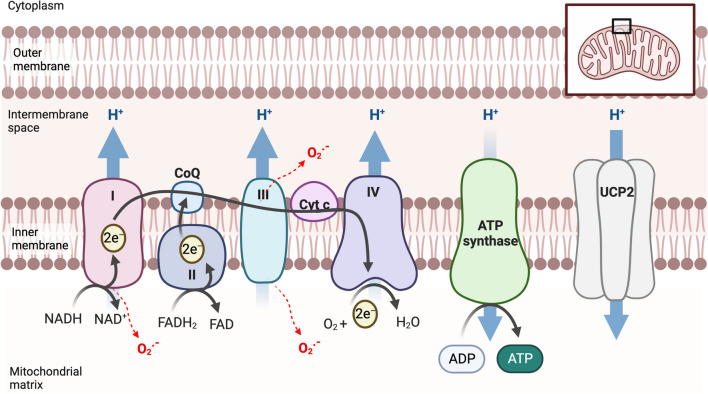
The ETC generates a proton gradient utilized by ATP synthase to generate ATP. UCP2 negates this gradient via mild uncoupling. Ucp2 indirectly controls ROS levels because when the proton gradient becomes too high electrons can escape from the ETC (red arrows) and react with oxygen to form reactive oxygen species. Ucp2 keeps the gradient at ideal levels, keeping the electrons moving through the pathway so they cannot form ROS

In cancer, the electron transport chain is highly stimulated, generating copious amounts of ATP to keep the tumor cells growing and expanding. However, mitochondria are one of the highest producers of ROS in the cell [123]; thus, the extended ETC use increases the level of ROS in the cell. If left unchecked, high levels of ROS can result in cell death. To evade this, cancer cells enact multiple methods of antioxidants, PDAC included. UCP2 upregulation is likely a way that PDAC cells manage ROS. By uncoupling the ATP generation from the ETC, UCP2 reduces the chance of electrons leaking out of the complexes and interacting with oxygen to form ROS (Fig. 2). Thus, although UCP2 overexpression slightly reduces the amount of ATP generated, it keeps ROS levels manageable, thus promoting cell growth and survival.

### Pharmacologic inhibition of UCP2

As discussed above, purine nucleotides are inhibitors of UCP2. However, these molecules, though potent, are not cell permeable, and thus are not feasible for use in the clinic. Genipin is a natural inhibitor of UCP2 extracted from the *Gardenia jasminoides* Ellis shrub in the Rubiaceae family. Fruits of this plant have been used in Asian communities as both a natural yellow, blue, and red colorant, as well as in traditional medicine to relax blood vessels and improve blood flow [124]. It has also been used to treat diabetes and neurological disorders [125]. Genipin forms from geniposide upon cleavage with β-glucosidase. Further reaction between genipin and amino acids or proteins results in the genipin blue colorant [124]. Genipin is a natural crosslinker, able to interact with proteins, collagen, gelatin, and chitosan, with low toxicity and high biocompatibility, making it a commonly used drug for drug delivery, despite lacking FDA-approval [124].

Despite its known crosslinking abilities, the exact mechanism of inhibition that genipin has on UCP2 was unknown, leading to its lack of popularity in classical medicine. It was first identified as a Ucp2 inhibitor in 2016 when insulin secretion was stimulated in pancreatic islet cells from wild-type mice with genipin treatment but not in those of mice lacking Ucp2. In this situation, genipin worked via a mechanism distinct of its crosslinking abilities [126]. Through synthesized genipin derivatives and UCP2 overexpression and silencing studies, the 1-OH group was determined to be the key site for its cytotoxic biological effect on PDAC cells [127]. Furthermore, at low concentrations, genipin binds arginine residues in the UCP funnel, leading to loss of proton leak abilities. At concentrations above 400 μM, however, there is increased nonspecific ion transport due to the formation of genipin-protein aggregates, leading to loss of specificity [125]. However, genipin was also found to inhibit the proton conductance of UCP1 and UCP3 in planar bilayer membranes, leading to speculation of genipin’s specificity [125]. Although the specificity of genipin has been questioned, knockout and silencing studies support the fact that this drug targets UCP2. Genipin inhibited proton leak in pancreatic islets, resulting in increased membrane potential and ATP levels, leading to K_ATP_ channel closing and the secretion of insulin [126]. These results were UCP2-dependent, as cells lacking UCP2 did not have these effects upon genipin treatment [126]. In addition, siRNA-mediate knockdown of UCP2 reversed the antiproliferative effects of genipin in pancreatic cancer [128], further supporting genipin specificity.

Due to its low cytotoxicity to normal cells and UCP2’s common overexpression in cancer, genipin’s anticancer effects have become an area of interest. In bladder cancer, genipin suppressed the growth of cancer cells, induced cell cycle arrest, promoted apoptosis, and inactivation of the PI3K/Akt signaling pathway, a vital pathway for bladder cancer [129]. In contrast, no impact to viability on non-malignant cells was observed [129]. In breast cancer, inhibition of UCP2 via genipin resulted in increased sensitivity to cisplatin and tamoxifen therapeutics, with additive effects on oxidative stress induction [130], and suppressed ROS, and reduced proliferation and invasion selectively in breast cancer compared to normal breast epithelial cells [22]. In drug-resistant leukemia cells, genipin treatment selectively sensitizes cells to cytotoxic drugs compared to drug-sensitive controls [131]. In addition, genipin was utilized to demonstrate that UCP2 is likely involved in the mechanism of action of cisplatin in colon cancer cells [132]. Finally, genipin reduced the glycolytic flux and mitochondrial OXPHOS in breast and colon cancer cells, resulting in a decrease in lactate release, OCR reduction, and ROS increase, and this effect was recapitulated by UCP2 knockdown [133]. Genipin has been implicated in autophagy suppression as well. Genipin inhibits the NLRP3 and NLRC4 inflammasomes via autophagy suppression, which are cytoplasmic complexes that control pro-inflammatory cytokine production. Thus, genipin may be a therapeutic option for inflammasome-related disorders such as colon cancers and type 2 diabetes [134].

The use of an uncoupling protein inhibitor brings up expected comparisons to mitochondrial uncoupler therapies. Mitochondrial uncoupling therapy may be beneficial in the treatment of obesity [135], fatty liver disease, and NASH [136], but studies have only recently extended to cancer. The earliest uncoupler, also known as a protonophore, 2,4-DNP (DNP) was first used in the 1930s as a weight-loss drug but was removed from the market by the FDA after it induced severe adverse events such as hyperthermia, agranulocytosis, and hepatotoxicity, limiting future clinical development of uncouplers [137]. Thus, classic protonophores such as DNP, FCCP (fluorinated hydrazonomalononitrile), and CCCP (chlorinated hydrazonomalononitrile) are more commonly used *in vitro* to investigate uncoupling effects on cells. However, some FDA-approved drugs are being evaluated for repurposing potential as uncouplers in cancer. Although no uncoupler therapies are currently approved for clinic use in any cancer, several clinical trials are ongoing. Niclosamide, a drug approved for tapeworm treatment, has shown antitumor effects in multiple cancer types by decreasing cell proliferation, inducing G1 cell cycle arrest, inhibiting mTOR, and inducing apoptosis [138]. However, niclosamide has poor water solubility and oral bioavailability [139], limiting its therapeutic potential. Indeed, a clinical trial exploring this oral administration of this drug in combination with enzalutamide in castration-resistant prostate cancer failed to improve patient outcome [140], but a phase II trial is currently ongoing exploring combinations with abiraterone and prednisone (NCT02807805). Nitazoxanide, an FDA-approved antiparasitic drug, has also shown uncoupling ability, resulting in AMPK activation, mTOR inhibition, G1 cell cycle arrest, and decrease ATP rates *in vitro* and is currently in an active clinical trial for advanced cancers in combination with irinotecan (NCT02366884). In addition to repurposing drugs, novel uncoupling drugs are also being synthesized. A novel niclosamide analog has shown efficacy in T cell leukemia by inhibiting proliferation, activating AMPK, and decreasing mTOR signaling [141].

In comparison with genipin, there seems to be less ROS generation with mitochondrial uncouplers, or it was not explored in depth in the papers mentioned here. Both uncouplers and genipin slow cell growth and induce apoptosis. Interestingly, while genipin seems to upregulate mTOR signaling and make PDAC more susceptible to combination therapy with everolimus [142], mitochondrial uncouplers seem to consistently downregulate mTOR signaling, highlighting the difference between these two distinct compound groups, and the exploration that is still needed into the mechanism of mitochondrial uncouplers. Indeed, where genipin seems to induce oxidative stress in cells, these uncouplers seem to induce dysfunction in other pathways involved in cell growth.

### UCP2 in PDAC

Inhibition of UCP2 as a therapeutic option in PDAC has become an increasing area of interest. The accepted role of UCP2 in PDAC is that it is downregulated during initiation of tumorigenesis to allow ROS accumulation and genomic instability, whereas it becomes overexpressed in later stages of tumorigenesis, contributing to tumor maintenance of high ATP generation, ROS protection, and treatment resistance [143], which has also been established in other cancers such as colorectal [23]. The combination of genipin with gemcitabine resulted in a significant increase of ROS production and synergized to inhibit cell proliferation compared to either treatment alone [144]. In addition, gemcitabine treatment induced UCP2 expression, suggesting that uncoupling may have a role in acquired resistance to gemcitabine treatment. In comparison, normal fibroblasts have drastically reduced response to this combination, suggesting a tumor-specific role for UCP2 in gemcitabine resistance, and the possibility of combination therapy with genipin [144]. UCP2 inhibition also slowed PDAC cell line growth, and the resulting rise in ROS-induced autophagy, synergizing with that induced by gemcitabine [142].

Glutamine metabolism inhibition also elevated ROS, opening up the option for combination therapy with genipin to further elevate ROS and potentially impact PDAC growth, as PDAC is glutamine addicted [12]. In addition, UCP2 enhances the Warburg effect, shifting PDAC cells away from OXPHOS metabolism and more towards glycolysis [61, 128, 145]; thus, UCP2 makes PDAC cells more dependent on glycolysis and sensitizes them to glycolytic inhibition via the UCP2 overexpression common in this cancer type [128]. UCP2 inhibition induces an upregulation of the Akt/mTOR pathway in a ROS-dependent manner and sensitized PDAC cells to the mTOR inhibitor everolimus [146]. Finally, UCP2 loss in PDAC cells reduced glutamine catabolism, increasing ROS, and inducing a shortage of aspartate, but only in KRAS mutant lines, supporting the fact that UCP2 is vital for glutamine-dependent tumors such as PDAC [16].

Although the combination of genipin with chemotherapies in PDAC has not been extensively explored beyond these few studies, recent exploration into combination therapy with genipin in other cancers provides insight that can be translated to PDAC. In most cases, UCP2 inhibition increases ROS, which improves therapeutic outcomes. For instance, uterine leiomyosarcoma (ULMS) upregulates UCP2 and has limited therapeutic options. However, treatment with the cardiac glycosides proscilla A and lanatoside downregulated UCP2 expression, resulting in antitumor effects via increased ROS, unveiling the opportunity for novel combination strategies with ROS-inducing therapies [147]. In breast cancer, UCP2 inhibition combined with cisplatin and tamoxifen [130], or trastuzumab [148] resulted in antitumor effects via increased oxidative stress, ROS generation, and apoptosis promotion. The combination of genipin and elesclomol synergistically increased ROS in lung cancer, while also reducing mitochondrial membrane potential and glucose uptake [149].

Given the ability of UCP2 inhibition to increase ROS, it is reasonable to assume that combining genipin with ROS-inducing chemotherapies would be a potent way to induce cancer cell death, which would be useful in pancreatic cancer. Indeed, chemotherapies such as topoisomerase inhibitors and platinum agents generate high levels of ROS [150]. There are two standard of care chemotherapy combinations used in PDAC: FOLFIRINOX and gemcitabine-nab-paclitaxel [151]. High ROS-inducing chemotherapies are central components of FOLFIRINOX, which utilizes irinotecan, a topoisomerase I inhibitor, and oxiplatin, a platinum agent. On the other hand, nucleotide analogues, such as 5-FU in FOLFIRINOX and gemcitabine in gemcitabine-nab-paclitaxel, may generate less ROS [150, 152]. Thus, given the known literature of ROS generation and genipin, the combination of FOLFIRINOX might be expected to synergize with genipin, while gemcitabine-nab-paclitaxel might not.

In addition, a role for Ucp2 in immunotherapy has recently been posited. In melanoma, the induction of Ucp2 expression promoted immune cell infiltration of the tumor and improved response to checkpoint inhibitor therapy. The Ucp2 expression levels correlated with CD8A mRNA, establishing a possible connection between Ucp2 and CD8^+^ T cell recruitment. It also correlated with interferon gamma expression which stimulated the tumor microenvironment, dendritic cell migration, and T cell recruitment. *In vivo*, Ucp2 induction with rosiglitazone prolonged the response to anti-PD1 immunotherapy [153]. While melanoma is a relatively “hot” tumor with high immune cell infiltration, it also upregulates UCP2, like PDAC [154]. PDAC is a characteristically “cold” tumor, with little immune cell infiltration [155], but further induction of UCP2 levels in the tumor may allow for increased infiltration of immune cells and better response to immunotherapy treatment.

An unexplored area in UCP2 biology in PDAC is the potential for combination therapy with genipin and radiation, as both modalities have been shown to increase ROS alone and radiation is one of the few treatment options available to PDAC patients. Thus it would be beneficial to explore if synergism occurs with these treatments together. Currently, very few studies have explored UCP2 and radiation, and none have been in PDAC. Cervical cancer cells had increased radiosensitivity upon UCP2 loss, resulting in reduced membrane potential, increased ROS levels, and cell cycle arrest, leading to apoptosis [156]. However, the impact of UCP2 loss or inhibition via genipin combined with radiation in PDAC cells has not been elucidated.

## UCP2 in mitochondrial dynamics

**Fig. 3:**
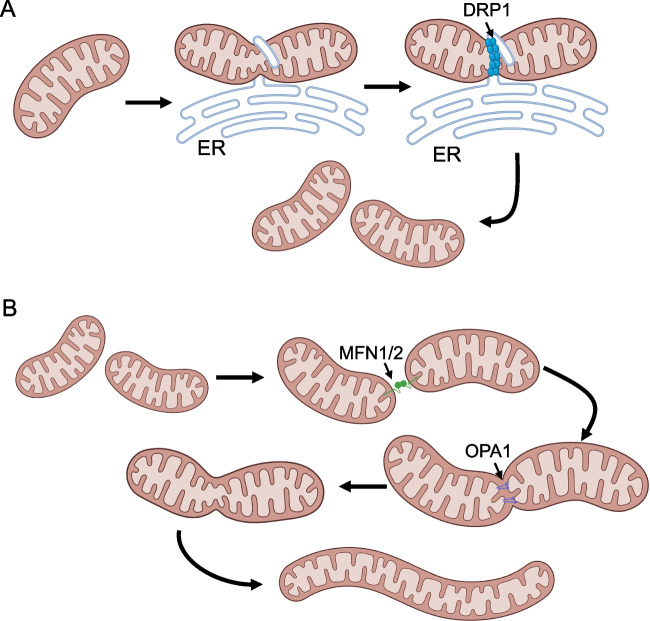
The process of mitochondrial dynamics is demonstrated by **A** mitochondrial fission: it is known that ER interacts with actin and mitochondria at division points. This results in the constriction of the mitochondria and facilitates DRP1 dephosphorylation and recruitment. DRP1 hydrolyzes GTP resulting in DRP1 tightening and the division of both mitochondrial membranes and **B** mitochondrial fusion: MFN1/2 coordination work to fuse the outer mitochondrial membrane, while OPA1 joins the inner mitochondrial membrane

Mitochondria are physically and metabolically dynamic organelles that alter their shape to optimize energy production for both normal and cancer cells via OXPHOS. Changes in mitochondrial morphology and function are enacted via the complementary processes of fission and fusion (Fig. 3). Mitochondrial fusion brings together two or more mitochondria, often during times of cellular stress [157] and is vital for mitigating this stress by mixing mitochondria content, providing a template to repair damaged mitochondrial DNA [158, 159]. On the other hand, mitochondrial fission involves the breakdown of larger mitochondria into smaller organelles. This process of fission is important for organelle quality control by removing damaged mitochondria via mitophagy and maintaining proper distribution of mitochondria throughout the cell during cell division [160–162]. There is specific machinery involved in both fusion and fission to maintain the proper balance of each process. In fusion, transmembrane GTPase proteins such as mitofusin (MFN) 1 and 2 [163–167], as well as optic atrophy protein 1 (OPA1) [168–171] work to fuse the outer and inner mitochondrial membranes, respectively, forming a syncytium of connected mitochondria [172, 173]. The main protein involved in fission is another GTPase known as dynamin-related protein 1 (DRP1) [174]. DRP1 is normally located in the cytosol but translocates to the mitochondria upon activating phosphorylation. At the mitochondria, DRP1 binds FIS1 and other receptors [175] on the outer mitochondria membrane. This induces DRP1 oligomerization and interaction with actin [176, 177] and the endoplasmic reticulum [178], resulting in GTP hydrolysis and complete mitochondrial membrane division [179].

The balance of fusion and fission is driven by the rapid and ever-changing needs of cells and is disrupted in a multitude of pathological conditions, including PDAC [180, 181]. In normal pancreatic cells, fusion is dominant, resulting in long and tubular mitochondrial morphology, while in PDAC the balance tips towards fission [182], resulting in a plethora of small, punctate mitochondria. However, the survival advantage that this grants PDAC cells is not entirely understood, especially considering that the loss of fusion or fission alone results in severe cellular defects [160, 183], and fission is normally a marker of cell stress [157].

Chemical uncouplers that result in unregulated and unrestrained depolarization of the inner mitochondrial membrane result in inhibition of fusion and the degradation of OPA1, leading to smaller mitochondria via unrestrained fission. In addition, fission was found to associate with a depolarized mitochondrial membrane state and increased ROS in pancreatic β cells [161]. Membrane depolarization also results in stabilization and cleavage of OMA-1, which cleaves OPA1, resulting in the inhibition of fusion in HeLa cells [184]. Thus, UCP2, with controlled and regulated uncoupling, may have a role in mitochondrial dynamics. Determining how two processes are linked together to drive the pathogenesis of disease will open avenues of therapeutic targeting. In neurons, UCP2 is required for glucose-induced Drp1-mediated fission, and UCP2 loss resulted in the loss of Drp1 phosphorylation and subsequent activation [185]. Loss of UCP2 in microglia also prevented the changes in mitochondrial dynamics usually see in high-fat diet feeding [186]. Deletion of UCP2 in ischemic mice resulted in increased ROS, elevation of fission proteins, and suppression of fusion proteins [187]. Acute kidney injury is associated with a high level of UCP2 expression and mitochondrial fission [188]. UCP2 loss exacerbated the fission and severity of kidney injury, and UCP2 overexpression protected against injury, shifting the cells back towards the proper mitochondrial dynamic balance with increased fusion [188]. After heat stroke, UCP2 was upregulated, and mitochondrial fission was induced in HUVECS [189]. This was recapitulated both with Drp1 pharmacological inhibition and UCP2 knockdown [189]. Thus, UCP2 and its connection to mitochondrial dynamics have been explored in multiple diseases. This connection has yet to be explored in PDAC but could potentially provide targeted therapy options for PDAC patients.

## Conclusion

**Fig. 4:**
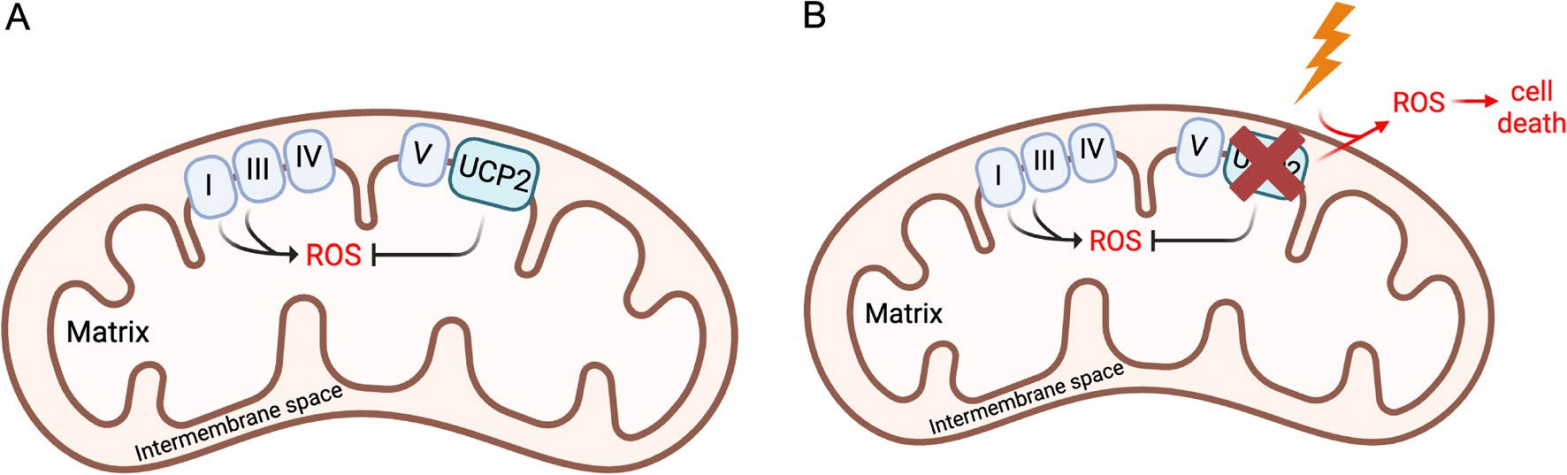
**a** Ucp2 modulates ROS levels via mild uncoupling. **b** Targeting Ucp2 via inhibition with genipin combined with radiation (lightning bolt) or other chemotherapies known to elevate ROS has the potential to synergize and overload cancer cells with ROS, leading to cell death

In summary, UCP2’s integral roles in cellular metabolism, ROS regulation, and mitochondrial dynamics underscore its significance in the pathophysiology of PDAC and other cancers. Further elucidation of its precise functions and regulatory mechanisms can pave the way for combined targeted therapeutic strategies utilizing UCP2 inhibitors such as genipin (Fig. 4) with radiation therapy to increase the treatment options for PDAC patients.
